# Impact of uncertain demand and channel power on dual channel supply chain decisions

**DOI:** 10.1371/journal.pone.0300386

**Published:** 2024-03-15

**Authors:** Liu-wei Zhao, Yin Zhao

**Affiliations:** 1 School of Management, Jiangsu University of Technology, Changzhou, Jiangsu, China; 2 School of Foreign Language, Jiangsu University of Technology, Changzhou, Jiangsu, China; University of Madeira / NOVA Lincs, PORTUGAL

## Abstract

The paper aims to conduct an analysis of pricing strategies in a dual channel supply chain under external uncertainty, utilizing Interval numbers theory and Game theory as the theoretical basis. The focus is on maximizing the expected profits of manufacturers and retailers. Four models are considered: centralized decision-making, manufacturer’s Stackelberg, retailer’s Stackelberg strategy, and vertical Nash model, with the decision variable being the product price. By solving the game model, the paper compares the optimal decisions under the four models and conducts sensitivity analysis to reflect the influence of key parameters and analyze their relationships. The ultimate goal is to optimize profits under various circumstances by adjusting market potential and price parameters to determine the best price level. The findings suggest that decision-maker’s risk indicators have a greater impact on decision results when market demand is less sensitive to price, and that the size of the market has a negative correlation with the impact of decision-maker’s risk indicators on decision results.

## 1 Introduction

In the past two decades, the retail industry has experienced substantial growth in online retail due to the rapid evolution of e-commerce. The number of enterprises establishing direct sales channels through e-commerce has increased, with an estimated 2 billion people worldwide engaging in online shopping in 2021, resulting in global electronic retail sales reaching $4.9 trillion. Major manufacturers such as Apple, Sony, and HP have incorporated online retail channels alongside their traditional retail channels to capitalize on the vast e-commerce market. Alibaba reported a transaction volume of 540.3 billion yuan. on November 11, 2021 alone, indicating a surge in online sales and motivating many manufacturers to establish online channels for direct product sales to consumers. Simultaneously, manufacturers like Dell and Lenovo, which previously relied primarily on online channels, are actively expanding their traditional retail channels to enhance product visibility and brand recognition. The introduction of online channels has led some consumers to shift from traditional distribution channels to online direct sales channels, prompting retailers operating through traditional distribution channels to readjust their product pricing to prevent a decline in profits. It is evident that product pricing is a critical concern for both manufacturing enterprises and their retailers engaged in dual-channel operations within the e-commerce environment.

The study of pricing strategies in dual channel supply chains has a rich and well-established history within academic research. Notable scholars such as Tsay and Agrawal, Cattani et al, and Kurata et al. have emphasized the pivotal role of channel pricing in achieving coordination within dual channels [1–3]. Through a dual-channel supply chain, manufacturers can directly engage with consumers and realize higher revenue [4]. The substantial transaction volume observed during online shopping festivals like Double Eleven each year indicates a significant market demand, reflecting the growing acceptance and recognition of pre-sales by consumers. Pre-sales, as an emerging sales model, not only generate anticipation for e-commerce shopping festivals in advance but also streamline bulk order processing to reduce operational costs. This efficient, large-scale, and zero inventory sales model has been widely adopted across industries due to its ability to reduce information uncertainty and increase product sales [5, 6]. The emergence of the new retail model, while enhancing customer satisfaction in shopping experiences, is expected to have a significant impact on offline retailers and lead to channel conflicts that impede sales performance growth. To address these challenges and facilitate effective cooperation between the two channels, it is imperative to establish reasonable contracts that promote coordination. Extensive research has been conducted by scholars on dual channel supply chains, with a focus on various aspects of channel pricing. For instance, Panda et al. and Wang et al. have examined the influence of manufacturer dominance and retailer dominance on pricing strategies within supply chains [7, 8]. Chen et al. have investigated decision-making problems related to pricing and quality in retail channels, direct sales channels, and dual channels [9]. Soleimani et al. have explored the pricing strategy problem in a dual channel supply chain, considering both centralized and decentralized decision-making approaches [10]. Qian et al. have examined the channel coordination problem in a two-level sustainable supply chain, analyzing environmental performance and channel profits under different types of contracts [11]. Additionally, Xu et al. and Cai have found that risk appetite, channel operating costs, and channel substitutability all impact channel coordination [12, 13]. Furthermore, studies have suggested using option contracts for risk sharing [14] and adjusting contract parameters for alleviating channel conflicts [15]. The proposals of linear batch discount contracts and revenue cost sharing contracts aim to coordinate channel relationships and achieve profit growth [16]. Li et al. have studied member pricing and green strategy issues in a dual channel supply chain for manufacturers producing green products, coordinating the dual channel green supply chain through contracts [17]. While these articles have delved into various aspects of dual channels, they have not fully considered the impact of demand uncertainty on coordination and pricing issues within dual channel supply chains.

Numerous empirical studies have shed light on the pivotal role of power dynamics within a supply chain in shaping pricing decisions. Chiang et al. investigated dual-channel pricing strategies, with manufacturers assuming leadership roles and retailers as followers, and found that equilibrium pricing is the most optimal strategy [18]. Fruchter and Tapiero delved into the varying levels of consumer acceptance towards online sales channels and concluded that consistent dual-channel pricing is the manufacturer’s most optimal strategy [19]. Yao and Liu explored Bertrand competition in a dual-channel system and provided optimal pricing strategies for both manufacturers and retailers [20]. Similarly, Cho and Tang compared and analyzed pricing and profit differences across scenarios where the manufacturer, retailer, or both have leadership status [21]. Furthermore, Cheng and Thorstenson utilized game theory and modeling methods to examine the impact of channel power and information structures on supply chain decision-making, revealing that as retailers’ power increases, manufacturers’ profits decline [22].

In recent years, there has been a growing interest in understanding optimal pricing strategies and decision-making processes in dual-channel systems and supply chains. Zhao et al. (2016) conducted a study on the influence of product reengineering strategy on member enterprises in closed-loop supply chains, analyzing the pricing strategy and supply chain system performance considering factors such as supplier strength, manufacturer strength, and the balance of power between the supplier and manufacturer [23]. The research findings indicated that the supply chain performance is optimal when there is a balance of power between suppliers and manufacturers, with a manufacturer-led supply chain being more favorable compared to a supplier-led supply chain. However, as powerful retailers like Gome and Suning gain prominence, the dynamics of the supply chain are shifting from being manufacturer-dominated to being retailer-dominated. This shift has prompted the need for a dual channel pricing model based on consumer utility selection, investigating the dual channel pricing strategies under different channel power structures. The primary focus will be on examining the impact of consumer perception of goods on the pricing strategy and profits of manufacturers and retailers in online channels. The findings of this study aim to provide decision-making support for manufacturing enterprises seeking to implement dual channel operations.

Furthermore, the performance of the entire supply chain is significantly affected by the stochastic nature of market demand, which is a result of the uncertainty of market information and the complexity of the decision-making environment. The imprecise nature of market information and the intricate decision-making environment make it challenging to accurately describe the market demand function using precise data. As a result, scholars have delved into various fuzzy set theories, such as interval-valued fuzzy sets, intuitionistic fuzzy sets, and interval-valued intuitionistic fuzzy sets, to tackle these challenges. These fuzzy set theories have been extended to theoretical research, enabling the resolution of multi-attribute decision problems and practical applications. For example, Yu and Liu investigated price competition and coordination among members of a mixed dual channel supply chain under random demand and joint promotion [14]. They derived a Nash equilibrium of prices and argued that the optimal price for online channels would reduce retailers’ efforts. Modak and Kelle highlighted the presence of uncertainty in the supply chain, which impacts the optimal order quantity, sales price, and delivery time [24]. Frascatore and Mahmoodi (2008) considered long-term contracts and penalty contracts in the context of random product demand [25]. They found that long-term contracts contribute to profit enhancement, while penalty contracts ensure that suppliers make decisions regarding production capacity levels. He et al. (2009) examined joint return strategies with revenue sharing contracts, return strategies with sales rebates and penalty contracts, and revenue sharing contracts with sales rebate and penalty contracts to address coordination issues in stochastic demand supply chains [26]. In addition, Pal et al. conducted a study on pricing decisions, levels of green innovation, and promotional efforts of participants in the context of green innovation [27]. They analyzed the impact of centralized Nash policies, manufacturer Stackelberg policies, and vertical Nash policies on these factors. Karimabadi et al. investigated the optimal decision-making process in a two-channel remanufacturing supply chain, examining wholesale price, retail price, direct price, and remanufacturing effort under both the centralized decision model and decentralized decision model [28]. The analysis in this study utilized fuzzy theory and game theory to investigate the impact of demand uncertainty on inter-firm competition in the supply chain. However, previous studies by Pal et al. and Karimabadi et al. did not take into account the attitudes or preferences of decision-makers. In practical supply chain management, decision-makers’ attitudes play a crucial role and can significantly influence final decision outcomes [27, 28]. Therefore, it is necessary to consider decision-makers’ attitudes towards supply chain risks and study the operational decision-making process of supply chain members and its evolution. Specifically, understanding how supply chain members make decisions under different competitive supply chain structures and how decision-makers’ risk attitudes influence these decisions is important. Additionally, in highly competitive industries such as home appliances, smartphones, electronic educational products, and machinery manufacturing, where manufacturers and retailers operate, there are numerous similar competitors. Hence, considering decision-makers’ risk attitudes is of great importance in researching this issue and holds practical significance.

Drawing on previous research, this paper utilizes interval numbers to quantify the demand function and integrates a mindset indicator to represent decision-makers’ risk attitudes. A dual channel pricing decision model is developed, taking into consideration varying power structures based on consumer utility selection. The main objective is to explore the impact of consumer perception of goods on online channels, demand uncertainty, and decision-maker risk preferences on manufacturers’ and retailers’ pricing decisions. This study aims to provide decision support for manufacturing enterprises in implementing dual channel operations. In contrast to existing literature, this paper’s main contributions are primarily evident in three key areas: (i) examining the risk attitudes of decision-makers and developing four distinct structured models of dual-channel supply chain competition, (ii) utilizing fuzzy theory to account for uncertain demand as a fuzzy variable, and (iii) investigating the dynamic evolution process of decision strategies for supply chain members in both horizontal and vertical dual competition scenarios. To address these issues, a Stackelberg game model with manufacturers as leaders and retailers as followers is established. The paper discusses centralized decision-making models, manufacturer Stackelberg strategies, retailer Stackelberg strategies, and vertical Nash strategy models using the reverse induction method. Furthermore, numerical examples are used to compare supply chain decision-making results and decision-maker risk attitudes under the four different rights structure models. Sensitivity analysis of interval numbers is conducted to demonstrate the impact of several variables on the decision-making results of dual-channel supply chains. Finally, management enlightenment is provided based on the findings.

This paper is organized as follows. In Section 2, we present definitions of the number of intervals and the ranking theory. Section 3 outlines the problem and introduces the developed model. Section 4 investigates optimal strategies under various power structures, such as Centralized decision making, Manufacturer Stackelberg Strategy, Retailer Stackelberg Strategy, and Vertical Nash strategy. Additionally, Section 4 compares Equilibrium Solutions with Different Rights Structures. In Section 5, we discuss the characteristics of the game model through numerical simulations, including numerical examples. Finally, in Section 6, we analyze the impact of variables on decision results and conduct sensitivity analysis.

## 2 Problem description and model construction

### 2.1 Research problem description

**Fig 1:**
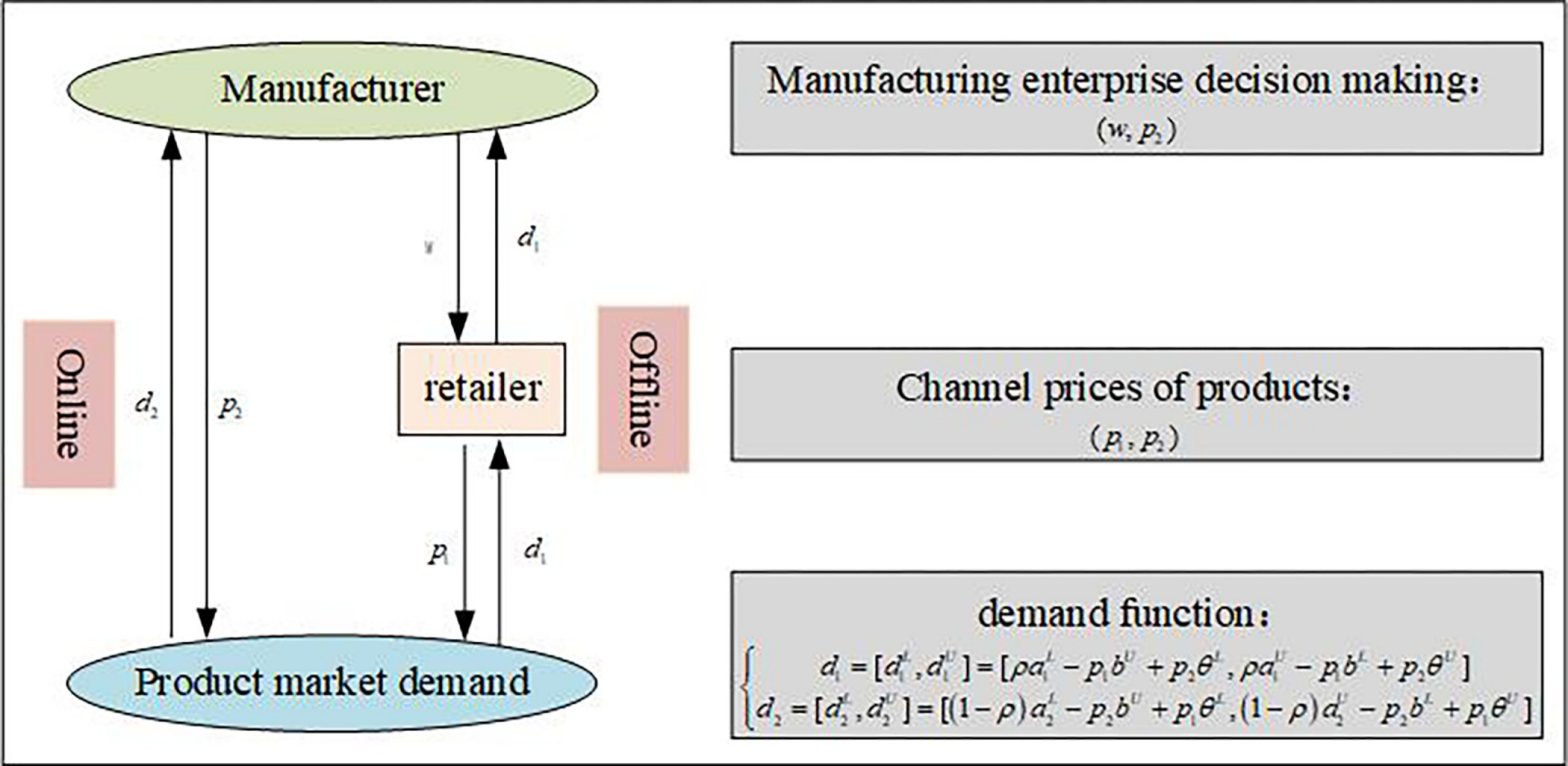
Schematic diagram of a dual channel supply chain network model under uncertain demand.

This article presents a comprehensive analysis of a dual channel supply chain network model with two levels. The model focuses on the production and distribution of a single type of product through both online and offline channels within a limited planning period *T*, as depicted in Fig 1. At the first level, manufacturers employ a traditional model of selling products to wholesalers, as well as a direct sales model through online channels, forming a dual channel sales strategy. At the second level, traditional offline retailers sell products to consumers. The decision-making model adopted in the supply chain leads to four distinct decision-making structures: Centralized decision making (CD), Manufacturer Stackelberg Strategy (MS), Retailer Stackelberg Strategy (RS), and Vertical Nash (VN) strategy. These structures represent different approaches to collaborative decision making and pricing strategies within the dual channel supply chain network. The implementation of centralized strategy management allows all participants to collectively optimize the profits of the entire supply chain. In the Manufacturer Stackelberg Strategy, manufacturers assume the role of leaders in determining wholesale prices, while in the Retailer Stackelberg Strategy, retailers take on this leadership role in setting retail prices. Additionally, when manufacturers and retailers possess equal control rights, they engage in a Vertical Nash game. Overall, this study provides valuable insights into the decision-making dynamics and pricing strategies within a dual channel supply chain network, offering important implications for businesses striving to expand their dual channel sales strategies.

For the purpose of scholarly research, it is posited that manufacturers and retailers possess equal capabilities in knowledge acquisition and absorption. The decision makers involved exhibit bounded rationality. The wholesale price of the manufacturer’s products is denoted as *w*>0, which is determined through negotiation between the manufacturer and retailer before the planning period and remains fixed during the planning period *T* with a certain time interval. Although the assumption of exogenous wholesale prices is primarily for analytical convenience, it holds true in certain scenarios [29, 30]. For instance, when manufacturers operate in highly competitive environments, they act as price takers, and wholesale prices are determined by market competition. Zhang et al. argued that in situations where controlling the pricing power of wholesale prices may reduce the willingness to share information and result in information leakage, manufacturers and retailers may commit to exogenous wholesale prices [31]. During the planning period *t*, consumers have the option to purchase products through online channels (online stores) or retail channels (physical stores), with the respective purchase prices denoted as *p*_1_ and *p*_2_. There exists a relationship, denoted as *w* = *kp*_2_ (0<*k*<1), between the wholesale price of manufacturers’ products through retail channels and the online direct sales price. Otherwise, the offline retail mechanism would be ineffective. The model also incorporates several other crucial decision variables, such as market size, product price, channel price sensitivity, and indicators of the decision-making mentality of manufacturers and retailers.

### 2.2 Model construction

In the realm of actual economic activities, economic activities are influenced by the intricate and constantly evolving market environment. The fluctuations in the external environment, decision-makers’ mindset, and competitors’ decision-making have all heightened the uncertainty surrounding product demand in the economic market. The intricate and ever-changing external environment often poses challenges in accurately quantifying the demand. Hence, this paper will utilize the interval number $a_{i}=[a_{i}^{L},a_{i}^{U}]$ (*a*_*i*_>0) to represent the potential demand of the projected market, $\sum_{i=1}a_{i}\leq$ the overall market size [32]; $b=[b^{L},b^{U}](0<b<1)$ represents the coefficient of price sensitivity for one’s own product; $\theta =[\theta^{L},\theta^{U}](0\leq \theta <1)$ represents the coefficient of price sensitivity for competitor channels, which reflects the degree of substitutability between products sold through two channels. Assuming *b*>*θ*, meaning that the coefficient of price sensitivity for one’s own product is greater than the coefficient of price sensitivity for competitor channels [33]. ρ (0 < ρ < 1) represents the proportion of demand that flows into traditional offline consumption channels, while the remaining (1−*ρ*) flows into online direct sales. Therefore, this paper adopts the demand model commonly employed in dual channel literature [34]. Assuming that under uncertain environmental constraints, the demand function can be expressed as follows:

$$d_{1}=[d_{1}^{L},d_{1}^{U}]=[\rho a_{1}^{L}-p_{1}b^{U}+p_{2}\theta^{L},\rho a_{1}^{U}-p_{1}b^{L}+p_{2}\theta^{U}],$$ (1)

$$d_{2}=[d_{2}^{L},d_{2}^{U}]=[(1-\rho )a_{2}^{L}-p_{2}b^{U}+p_{1}\theta^{L},(1-\rho )a_{2}^{U}-p_{2}b^{L}+p_{1}\theta^{U}].$$ (2)

Manufacturers and retailers face the complex task of formulating and implementing dynamic pricing strategies to maximize their profits over a specific time frame. This issue of profit maximization can be analyzed by examining the profit function of each party, taking into account their revenues and costs. This study centers on the management of a dual channel supply chain, assuming that the products are uniform with no differences, and that the marginal production cost is negligible for research purposes.

#### 2.2.1 Manufacturer’s profit function

Manufacturers distribute their products through both offline retail channels and online channels. Based on the given assumptions, the demand for offline retail and online channels is represented by *d*_1_ and *d*_2_, respectively. The wholesale prices are *w* and the online prices are *p*_2_. Consequently, the manufacturer’s revenue can be calculated as $d_{1}w+d_{2}p_{2}=d_{1}kp_{2}+d_{2}p_{2}$ (*w* = *kp*_2_). Therefore, the total profit of the manufacturer can be expressed in the following form:

$$\pi_{m}=(k[\rho a_{1}^{L}-p_{1}b^{U}+p_{2}\theta^{L},\rho a_{1}^{U}-p_{1}b^{L}+p_{2}\theta^{U}]p_{2}+[(1-\rho )a_{2}^{L}-p_{2}b^{U}+p_{1}\theta^{L},(1-\rho )a_{2}^{U}-p_{2}b^{L}+p_{1}\theta^{U}])p_{2}.$$ (3)

#### 2.2.2 Retailer’s profit function

Retailers sell products through retail channels. The demand function for retail channels is represented by *d*_1_, and the retail price is denoted as *p*_1_>0. Therefore, the total profit of retailers can be expressed in the following form:

$$\pi_{r}=d_{1}(p_{1}-w)=[\rho a_{1}^{L}-p_{1}b^{U}+p_{2}\theta^{L},\rho a_{1}^{U}-p_{1}b^{L}+p_{2}\theta^{U}](p_{1}-kp_{2}).$$ (4)

Hence, the overall profitability of the supply chain can be derived by utilizing Eqs (3) and (4) in the following manner:

$$\pi_{c}=[\rho a_{1}^{L}-p_{1}b^{U}+p_{2}\theta^{L},\rho a_{1}^{U}-p_{1}b^{L}+p_{2}\theta^{U}]p_{1}+[(1-\rho )a_{2}^{L}-p_{2}b^{U}+p_{1}\theta^{L},(1-\rho )a_{2}^{U}-p_{2}b^{L}+p_{1}\theta^{U}]p_{2}.$$ (5)

## 3 Analysis of optimal strategies under different power structures

According to the theory of interval ranking [35, 36], the ranking indicators of the demand function can be expressed in the following form:

$$d_{1}(\beta )=\rho a_{1}^{L}-p_{1}b^{U}+p_{2}\theta^{L}+\beta_{1}(\rho (a_{1}^{U}-a_{1}^{L})-p_{1}(b^{U}-b^{L})+p_{2}(\theta^{U}-\theta^{L})),$$ (6)

$$d_{2}(\beta )=(\left(1-\rho )a_{2}^{L}-p_{2}b^{U}+p_{1}\theta^{L}+\beta_{2}(\left(1-\rho )(a_{2}^{U}-a_{2}^{L})-p_{2}(b^{U}-b^{L})+p_{1}(\theta^{U}-\theta^{L}\right)\right)),$$ (7)

where *β*_*i*_∈[0,1] is the mentality indicator coefficient of decision-makers participating in the supply chain. If *β*_*i*_ = 0.5, it represents a state of mind where decision-makers maintain a moderate attitude. If *β*_*i*_ = 0, it indicates that the decision-maker holds a cautious or pessimistic attitude. If *β*_*i*_ = 1, it indicates that the decision-maker holds an optimistic and radical attitude.

Therefore, the interval ranking index Eqs (6) and (7) of demand can be incorporated into the profit Eqs (3), (4), and (5) to obtain the interval ranking index functions of manufacturers, retailers, and the total profit of the supply chain as follows:

$$\pi_{m}=(\begin{array}{l} k(\rho (a_{1}^{L}+\beta_{1}(a_{1}^{U}-a_{1}^{L}))-p_{1}(b^{U}+\beta_{1}(b^{U}-b^{L}))+p_{2}(\theta^{L}+\beta_{1}(\theta^{U}-\theta^{L})))\\ +(1-\rho )(a_{2}^{L}+\beta_{2}(a_{2}^{U}-a_{2}^{L}))-p_{2}(b^{U}+\beta_{2}(b^{U}-b^{L}))+p_{1}(\theta^{L}+\beta_{2}(\theta^{U}-\theta^{L})) \end{array})p_{2},$$ (8)

$$\pi_{r}=(\rho (a_{1}^{L}+\beta_{1}(a_{1}^{U}-a_{1}^{L}))-p_{1}(b^{U}+\beta_{1}(b^{U}-b^{L}))+p_{2}(\theta^{L}+\beta_{1}(\theta^{U}-\theta^{L})))(p_{1}-kp_{2}),$$ (9)

$$\begin{array}{l} \pi_{c}=(\rho (a_{1}^{L}+\beta_{1}(a_{1}^{U}-a_{1}^{L}))-p_{1}(b^{U}+\beta_{1}(b^{U}-b^{L}))+p_{2}(\theta^{L}+\beta_{1}(\theta^{U}-\theta^{L})))p_{1}\\ \;+(\left(1-\rho )(a_{2}^{L}+\beta_{2}(a_{2}^{U}-a_{2}^{L}))-p_{2}(b^{U}+\beta_{2}(b^{U}-b^{L}))+p_{1}(\theta^{L}+\beta_{2}(\theta^{U}-\theta^{L})\right))p_{2} \end{array}.$$ (10)

The following discussion aims to address the issue of optimal dynamic pricing in the context of centralized supply chain decision-making (CD), Manufacturer Stackelberg (MS), Retailer Stackelberg strategy (RS), and Vertical Nash (VN). It will compare and analyze the optimal dynamic pricing strategies under different power structures of manufacturers and retailers.

### 3.1 CD decision structure

In the context of CD decision-making, a consortium of supply chain networks with diverse requirements convene to achieve cost savings through collaborative production, procurement, and sales activities. The implementation of a centralized strategy management approach facilitates collective optimization of the overall chain’s profitability. Under this strategy, all participants engage in joint decision-making processes, thereby enabling them to attain maximum profit levels.

Find the first-order conditions for the total profit function Eq (10) of the supply chain regarding the retailer’s retail price *p*_1_ and the manufacturer’s online sales price *p*_2_, and organize them to obtain the following equation:

$$\partial \pi_{c}/\partial p_{1}=\rho (a_{1}^{L}+\beta_{1}(a_{1}^{U}-a_{1}^{L}))-2p_{1}(b^{U}+\beta_{1}(b^{U}-b^{L}))+p_{2}(\left(\theta^{L}+\beta_{1}(\theta^{U}-\theta^{L}))+(\theta^{L}+\beta_{2}(\theta^{U}-\theta^{L})\right)),$$ (11)

$$\partial \pi_{c}/\partial p_{2}=(1-\rho )(a_{2}^{L}+\beta_{2}(a_{2}^{U}-a_{2}^{L}))-2p_{2}(b^{U}+\beta_{2}(b^{U}-b^{L}))+p_{1}(\left(\theta^{L}+\beta_{1}(\theta^{U}-\theta^{L}))+(\theta^{L}+\beta_{2}(\theta^{U}-\theta^{L})\right)).$$ (12)

Let *∂π*_*c*_/*∂p*_*i*_ = 0, by combining Eqs (11) and (12), the strategy under centralized supply chain decision-making can be obtained and organized as follows:

$$p_{11}^{*}=\frac{2\rho (a_{1}^{L}+\beta_{1}(a_{1}^{U}-a_{1}^{L}))(b^{U}+\beta_{2}(b^{U}-b^{L}))+(1-\rho )(\left(\theta^{L}+\beta_{1}(\theta^{U}-\theta^{L}))+(\theta^{L}+\beta_{2}(\theta^{U}-\theta^{L})\right))(a_{2}^{L}+\beta_{2}(a_{2}^{U}-a_{2}^{L}))}{4(b^{U}+\beta_{1}(b^{U}-b^{L}))(b^{U}+\beta_{2}(b^{U}-b^{L}))-{\left(\left(\theta^{L}+\beta_{1}(\theta^{U}-\theta^{L}))+(\theta^{L}+\beta_{2}(\theta^{U}-\theta^{L})\right)\right)}^{2}},$$ (13)

$$p_{12}^{*}=\frac{\left(2(1-\rho )(b^{U}+\beta_{1}(b^{U}-b^{L}))(a_{2}^{L}+\beta_{2}(a_{2}^{U}-a_{2}^{L}))+\rho (a_{1}^{L}+\beta_{1}(a_{1}^{U}-a_{1}^{L}))(\left(\theta^{L}+\beta_{1}(\theta^{U}-\theta^{L}))+(\theta^{L}+\beta_{2}(\theta^{U}-\theta^{L})\right))\right)}{4(b^{U}+\beta_{1}(b^{U}-b^{L}))(b^{U}+\beta_{2}(b^{U}-b^{L}))-{\left(\left(\theta^{L}+\beta_{1}(\theta^{U}-\theta^{L}))+(\theta^{L}+\beta_{2}(\theta^{U}-\theta^{L})\right)\right)}^{2}}.$$ (14)

The only standing point for Eqs (11) and (12) is $M(p_{11}^{*},p_{12}^{*})$. Because $p_{11}^{*}$ and $p_{12}^{*}$ have certain economic practical implications, and since $p_{11}^{*}$ and $p_{12}^{*}$ are greater than 0, it is known that $4(b^{U}+\beta_{1}(b^{U}-b^{L}))(b^{U}+\beta_{2}(b^{U}-b^{L}))$ > ${\left(\left(\theta^{L}+\beta_{1}(\theta^{U}-\theta^{L}))+(\theta^{L}+\beta_{2}(\theta^{U}-\theta^{L})\right)\right)}^{2}$ is constant.

Next, we will prove that the unique stationary point $M(p_{11}^{*},p_{12}^{*})$ of Eqs (11) and (12) is the optimal strategy. Firstly, the Hesse matrix of the total profit function *π*_*c*_ of the supply chain is obtained by calculation as follows:

$$H_{1}=(\begin{array}{cc} \partial^{2}\pi_{c}/\partial p_{1}^{2} & \partial^{2}\pi_{c}/\partial p_{1}\partial p_{2}\\ \partial^{2}\pi_{c}/\partial p_{2}\partial p_{1} & \partial^{2}\pi_{c}/\partial p_{2}^{2} \end{array})=(\begin{array}{cc} -2(b^{U}+\beta_{1}(b^{U}-b^{L})) & \left(\theta^{L}+\beta_{1}(\theta^{U}-\theta^{L}))+(\theta^{L}+\beta_{2}(\theta^{U}-\theta^{L})\right)\\ \left(\theta^{L}+\beta_{1}(\theta^{U}-\theta^{L}))+(\theta^{L}+\beta_{2}(\theta^{U}-\theta^{L})\right) & -2(b^{U}+\beta_{2}(b^{U}-b^{L})) \end{array}).$$ (15)

It can be seen that the Hesse matrix of the total profit function *π*_*c*_ in the supply chain is independent of the values of $p_{11}^{*}$ and $p_{12}^{*}$. The Hesse matrix obtained at point $M(p_{11}^{*},p_{12}^{*})$ is in the following form:

$$H_{1}(M)=H_{1}=(\begin{array}{cc} -2(b^{U}+\beta_{1}(b^{U}-b^{L})) & \left(\theta^{L}+\beta_{1}(\theta^{U}-\theta^{L}))+(\theta^{L}+\beta_{2}(\theta^{U}-\theta^{L})\right)\\ \left(\theta^{L}+\beta_{1}(\theta^{U}-\theta^{L}))+(\theta^{L}+\beta_{2}(\theta^{U}-\theta^{L})\right) & -2(b^{U}+\beta_{2}(b^{U}-b^{L})) \end{array}).$$ (16)

According to Eq (16), it can be seen that the main and sub equations Δ_1_ = −2*Z*_1_<0 and $\Delta_{2}=|H_{1}|=4Z_{1}Z_{2}-{\left(Y_{1}+Y_{2}\right)}^{2}>0$ of the Hesse matrix *H*_1_ at the unique stagnation point $M(p_{11}^{*},p_{12}^{*})$ are constant. It can be inferred that the Hesse matrix *H*_1_ is negatively definite at the stagnation point $M(p_{11}^{*},p_{12}^{*})$, and the total profit function *π*_*c*_ of the supply chain will be the maximum at $M(p_{11}^{*},p_{12}^{*})$, which is the optimal value strategy for centralized decision-making in the supply chain.

### 3.2 MS decision structure

In the realm of strategic decision-making, the Stackelberg game stands as a prominent model wherein a leading company takes the initiative in setting a strategy, followed by subsequent actions from other companies. This study delves into the dynamics of this game, with a focus on the manufacturer as the pivotal decision-maker for the retailer. The retailer, in turn, adjusts pricing variables to optimize profits based on the manufacturer’s decisions. As such, the manufacturer wields significant influence over the market, with retailers aligning their strategies accordingly. This hierarchical structure underscores the asymmetry of power and decision-making within the supply chain.

It is easy to verify that the profit function *π*_*r*_ of a retailer is a concave function ($\partial^{2}\pi_{r}/\partial p_{1}^{2}=-2Z_{1}<0$) related to *p*_1_. The first order condition for obtaining the profit function of retailers with respect to *p*_1_ is as follows:

$$\partial \pi_{r}/\partial p_{1}=\rho (a_{1}^{L}+\beta_{1}(a_{1}^{U}-a_{1}^{L}))+p_{2}(\theta^{L}+\beta_{1}(\theta^{U}-\theta^{L}))+(b^{U}+\beta_{1}(b^{U}-b^{L}))(kp_{2}-2p_{1}).$$ (17)

Let ∂*π*_*r*_/∂*p*_1_ = 0, we can obtain:

$$p_{1}=\frac{\rho (a_{1}^{L}+\beta_{1}(a_{1}^{U}-a_{1}^{L}))+p_{2}(\left(\theta^{L}+\beta_{1}(\theta^{U}-\theta^{L}))+k(b^{U}+\beta_{1}(b^{U}-b^{L})\right))}{2(b^{U}+\beta_{1}(b^{U}-b^{L}))}.$$ (18)

By incorporating Eq (18) into Eq (8) of the manufacturer’s profit function, the following form can be obtained:

$$\pi_{m}=\frac{p_{2}(\begin{array}{l} 2(1-\rho )(a_{2}^{L}+\beta_{2}(a_{2}^{U}-a_{2}^{L}))(b^{U}+\beta_{1}(b^{U}-b^{L}))+\rho (a_{1}^{L}+\beta_{1}(a_{1}^{U}-a_{1}^{L}))(\left(\theta^{L}+\beta_{2}(\theta^{U}-\theta^{L}))+k(b^{U}+\beta_{1}(b^{U}-b^{L})\right))\\ +p_{2}\left(\begin{array}{l} \left(\theta^{L}+\beta_{1}(\theta^{U}-\theta^{L}))(\left(\theta^{L}+\beta_{2}(\theta^{U}-\theta^{L}))+k(b^{U}+\beta_{1}(b^{U}-b^{L})\right)\right)\\ +(b^{U}+\beta_{1}(b^{U}-b^{L}))(k(\theta^{L}+\beta_{2}(\theta^{U}-\theta^{L}))-k^{2}(b^{U}+\beta_{1}(b^{U}-b^{L}))-2(b^{U}+\beta_{2}(b^{U}-b^{L}))) \end{array}\right) \end{array})}{2(b^{U}+\beta_{1}(b^{U}-b^{L}))}.$$ (19)

Therefore, the first-order condition of the manufacturer’s profit function Eq (19) regarding *p*_2_ can be obtained as follows:

$$\frac{\partial \pi_{m}}{\partial p_{2}}=\frac{\left(\begin{array}{l} 2(1-\rho )(a_{2}^{L}+\beta_{2}(a_{2}^{U}-a_{2}^{L}))(b^{U}+\beta_{1}(b^{U}-b^{L}))+\rho (a_{1}^{L}+\beta_{1}(a_{1}^{U}-a_{1}^{L}))(\left(\theta^{L}+\beta_{2}(\theta^{U}-\theta^{L}))+k(b^{U}+\beta_{1}(b^{U}-b^{L})\right))\\ +2p_{2}\left(\begin{array}{l} \left(\theta^{L}+\beta_{1}(\theta^{U}-\theta^{L}))(\left(\theta^{L}+\beta_{2}(\theta^{U}-\theta^{L}))+k(b^{U}+\beta_{1}(b^{U}-b^{L})\right)\right)\\ +(b^{U}+\beta_{1}(b^{U}-b^{L}))(k(\theta^{L}+\beta_{2}(\theta^{U}-\theta^{L}))-k^{2}(b^{U}+\beta_{1}(b^{U}-b^{L}))-2(b^{U}+\beta_{2}(b^{U}-b^{L}))) \end{array}\right) \end{array}\right)}{2(b^{U}+\beta_{1}(b^{U}-b^{L}))}.$$ (20)

Let ∂*π*_*m*_/∂*p*_2_ = 0, we can obtain:

$$p_{22}^{*}=\frac{-2(1-\rho )(a_{2}^{L}+\beta_{2}(a_{2}^{U}-a_{2}^{L}))(b^{U}+\beta_{1}(b^{U}-b^{L}))-\rho (a_{1}^{L}+\beta_{1}(a_{1}^{U}-a_{1}^{L}))(\left(\theta^{L}+\beta_{2}(\theta^{U}-\theta^{L}))+k(b^{U}+\beta_{1}(b^{U}-b^{L})\right))}{2(\begin{array}{l} \left(\theta^{L}+\beta_{1}(\theta^{U}-\theta^{L}))(\left(\theta^{L}+\beta_{2}(\theta^{U}-\theta^{L}))+k(b^{U}+\beta_{1}(b^{U}-b^{L})\right)\right)\\ +(b^{U}+\beta_{1}(b^{U}-b^{L}))(k(\theta^{L}+\beta_{2}(\theta^{U}-\theta^{L}))-k^{2}(b^{U}+\beta_{1}(b^{U}-b^{L}))-2(b^{U}+\beta_{2}(b^{U}-b^{L}))) \end{array})}.$$ (21)

Since $p_{22}^{*}$ is greater than 0, it is known that

$(b^{U}+\beta_{1}(b^{U}-b^{L}))(\begin{array}{l} k(\theta^{L}+\beta_{2}(\theta^{U}-\theta^{L}))-k^{2}(b^{U}+\beta_{1}(b^{U}-b^{L}))\\ -2(b^{U}+\beta_{2}(b^{U}-b^{L})) \end{array})+(\theta^{L}+\beta_{1}(\theta^{U}-\theta^{L}))(\begin{array}{l} (\theta^{L}+\beta_{2}(\theta^{U}-\theta^{L}))+\\ k(b^{U}+\beta_{1}(b^{U}-b^{L})) \end{array})<0$ is constant. By introducing Eq (21) into Eq (18), it can be solved that

$$p_{21}^{*}=\frac{\left(\begin{array}{l} \rho (a_{1}^{L}+\beta_{1}(a_{1}^{U}-a_{1}^{L}))\left(\begin{array}{l} \left(b^{U}+\beta_{1}(b^{U}-b^{L}))(k(\theta^{L}+\beta_{2}(\theta^{U}-\theta^{L}))-3k^{2}(b^{U}+\beta_{1}(b^{U}-b^{L}))-4(b^{U}+\beta_{2}(b^{U}-b^{L}))\right)\\ +(\theta^{L}+\beta_{1}(\theta^{U}-\theta^{L}))(\left(\theta^{L}+\beta_{2}(\theta^{U}-\theta^{L}))+k(b^{U}+\beta_{1}(b^{U}-b^{L})\right)) \end{array}\right)\\ +2(1-\rho )(a_{2}^{L}+\beta_{2}(a_{2}^{U}-a_{2}^{L}))(b^{U}+\beta_{1}(b^{U}-b^{L}))(\left(\theta^{L}+\beta_{1}(\theta^{U}-\theta^{L}))+k(b^{U}+\beta_{1}(b^{U}-b^{L})\right)) \end{array}\right)}{4(b^{U}+\beta_{1}(b^{U}-b^{L}))(\begin{array}{l} \left(\theta^{L}+\beta_{1}(\theta^{U}-\theta^{L}))(\left(\theta^{L}+\beta_{2}(\theta^{U}-\theta^{L}))+k(b^{U}+\beta_{1}(b^{U}-b^{L})\right)\right)\\ +(b^{U}+\beta_{1}(b^{U}-b^{L}))(k(\theta^{L}+\beta_{2}(\theta^{U}-\theta^{L}))-k^{2}(b^{U}+\beta_{1}(b^{U}-b^{L}))-2(b^{U}+\beta_{2}(b^{U}-b^{L}))) \end{array})}.$$ (22)

Because $\frac{\partial^{2}\pi_{m}}{\partial p_{2}^{2}}=\frac{\left(\theta^{L}+\beta_{1}(\theta^{U}-\theta^{L}))(\left(\theta^{L}+\beta_{2}(\theta^{U}-\theta^{L}))+k(b^{U}+\beta_{1}(b^{U}-b^{L})\right))+(b^{U}+\beta_{1}(b^{U}-b^{L}))(k(\theta^{L}+\beta_{2}(\theta^{U}-\theta^{L}))-k^{2}(b^{U}+\beta_{1}(b^{U}-b^{L}))-2(b^{U}+\beta_{2}(b^{U}-b^{L}))\right)}{\left(b^{U}+\beta_{1}(b^{U}-b^{L})\right)}<0$ was established. Therefore, $p_{21}^{*}$ and $p_{22}^{*}$ are the maximum values, which is the optimal value strategy of the manufacturer’s Stackelberg strategy.

### 3.3 RS decision structure

In the RS Decision Structure, retailers are responsible for determining pricing variables in order to optimize the manufacturer’s decision variables and ultimately maximize profits. Manufacturers then make decisions regarding wholesale prices and online channel prices based on the decisions made by retailers. As a result, retailers wield significant control over the market, with manufacturers subsequently aligning their decisions with those of the retailers.

Firstly, the first-order condition for the manufacturer’s profit Eq (8) regarding *p*_2_ is as follows:

$$\partial \pi_{m}/\partial p_{2}=(\begin{array}{l} \left(1-\rho )(a_{2}^{L}+\beta_{2}(a_{2}^{U}-a_{2}^{L}))+p_{1}(\theta^{L}+\beta_{2}(\theta^{U}-\theta^{L}))-kp_{1}(b^{U}+\beta_{1}(b^{U}-b^{L})\right)\\ +2p_{2}(k(\theta^{L}+\beta_{1}(\theta^{U}-\theta^{L}))-(\theta^{L}+\beta_{2}(\theta^{U}-\theta^{L})))+k\rho (a_{1}^{L}+\beta_{1}(a_{1}^{U}-a_{1}^{L})) \end{array}).$$ (23)

Let ∂*π*_*m*_/∂*p*_2_ = 0, we can obtain:

$$p_{2}=\frac{\left(-(1-\rho )(a_{2}^{L}+\beta_{2}(a_{2}^{U}-a_{2}^{L}))-p_{1}(\left(\theta^{L}+\beta_{2}(\theta^{U}-\theta^{L}))-k(b^{U}+\beta_{1}(b^{U}-b^{L})\right))-k\rho (a_{1}^{L}+\beta_{1}(a_{1}^{U}-a_{1}^{L}))\right)}{2(k(\theta^{L}+\beta_{1}(\theta^{U}-\theta^{L}))-(b^{U}+\beta_{2}(b^{U}-b^{L})))}.$$ (24)

If Eq (24) is the manufacturer’s optimal equilibrium solution, it first satisfies *p*_2_>0 and condition $\partial^{2}\pi_{m}/\partial p_{2}^{2}=2(k(b^{U}+\beta_{1}(b^{U}-b^{L}))-(b^{U}+\beta_{2}(b^{U}-b^{L})))<0$, that is, $-(1-\rho )(a_{2}^{L}+\beta_{2}(a_{2}^{U}-a_{2}^{L}))-k\rho (a_{1}^{L}+\beta_{1}(a_{1}^{U}-a_{1}^{L}))$ $-p_{1}(\left(\theta^{L}+\beta_{2}(\theta^{U}-\theta^{L}))-k(b^{U}+\beta_{1}(b^{U}-b^{L})\right))<0$.

Bring Eq (24) into Eq (9) of the retailer’s profit function, the following form can be obtained:

$$\pi_{r}=\frac{\left(\begin{array}{l} p_{1}(\begin{array}{l} k^{2}(b^{U}+\beta_{1}(b^{U}-b^{L}))+2(b^{U}+\beta_{2}(b^{U}-b^{L}))\\ -2k(\theta^{L}+\beta_{1}(\theta^{U}-\theta^{L}))-k(\theta^{L}+\beta_{2}(\theta^{U}-\theta^{L})) \end{array})\\ -k(1-\rho )(a_{2}^{L}+\beta_{2}(a_{2}^{U}-a_{2}^{L}))-k^{2}\rho (a_{1}^{L}+\beta_{1}(a_{1}^{U}-a_{1}^{L})) \end{array})(\begin{array}{l} \left(1-\rho )(a_{2}^{L}+\beta_{2}(a_{2}^{U}-a_{2}^{L}))(\theta^{L}+\beta_{1}(\theta^{U}-\theta^{L})\right)\\ +p_{1}(\theta^{L}+\beta_{1}(\theta^{U}-\theta^{L}))(\left(\theta^{L}+\beta_{2}(\theta^{U}-\theta^{L}))+k(b^{U}+\beta_{1}(b^{U}-b^{L})\right))\\ -2\rho (a_{1}^{L}+\beta_{1}(a_{1}^{U}-a_{1}^{L}))(k(\theta^{L}+\beta_{1}(\theta^{U}-\theta^{L}))-(b^{U}+\beta_{2}(b^{U}-b^{L})))\\ -2p_{1}(b^{U}+\beta_{1}(b^{U}-b^{L}))(b^{U}+\beta_{2}(b^{U}-b^{L}))+k\rho (\theta^{L}+\beta_{1}(\theta^{U}-\theta^{L}))(a_{1}^{L}+\beta_{1}(a_{1}^{U}-a_{1}^{L})) \end{array}\right)}{4{\left(k(\theta^{L}+\beta_{1}(\theta^{U}-\theta^{L}))-(b^{U}+\beta_{2}(b^{U}-b^{L}))\right)}^{2}}.$$ (25)

Therefore, the first-order condition for the retailer profit function Eq (25) regarding *p*_1_ be obtained as follows:

$$\frac{\partial \pi_{r}}{\partial p_{1}}=-\frac{\left(\begin{array}{l} \left(\begin{array}{l} -(\theta^{L}+\beta_{1}(\theta^{U}-\theta^{L}))\left(\begin{array}{l} \left(\theta^{L}+\beta_{2}(\theta^{U}-\theta^{L})\right)\\ +k(b^{U}+\beta_{1}(b^{U}-b^{L})) \end{array}\right)\\ +2(b^{U}+\beta_{1}(b^{U}-b^{L}))(b^{U}+\beta_{2}(b^{U}-b^{L})) \end{array}\right)\left(\begin{array}{l} p_{1}\left(\begin{array}{l} -2k(\theta^{L}+\beta_{1}(\theta^{U}-\theta^{L}))-k(\theta^{L}+\beta_{2}(\theta^{U}-\theta^{L}))\\ +k^{2}(b^{U}+\beta_{1}(b^{U}-b^{L}))+2(b^{U}+\beta_{2}(b^{U}-b^{L})) \end{array}\right)\\ +k(-1+\rho )(a_{2}^{L}+\beta_{2}(a_{2}^{U}-a_{2}^{L}))-k^{2}\rho (a_{1}^{L}+\beta_{1}(a_{1}^{U}-a_{1}^{L})) \end{array}\right)+\\ \left(\begin{array}{l} k^{2}(b^{U}+\beta_{1}(b^{U}-b^{L}))\\ -k(\theta^{L}+\beta_{2}(\theta^{U}-\theta^{L}))\\ +2(b^{U}+\beta_{2}(b^{U}-b^{L}))\\ -2k(\theta^{L}+\beta_{1}(\theta^{U}-\theta^{L})) \end{array}\right)\left(\begin{array}{l} k(-1+\rho )(a_{2}^{L}+\beta_{2}(a_{2}^{U}-a_{2}^{L}))(\theta^{L}+\beta_{1}(\theta^{U}-\theta^{L}))\\ -p_{1}(\theta^{L}+\beta_{1}(\theta^{U}-\theta^{L}))(\theta^{L}+\beta_{2}(\theta^{U}-\theta^{L}))-kp_{1}(\theta^{L}+\beta_{1}(\theta^{U}-\theta^{L}))(b^{U}+\beta_{1}(b^{U}-b^{L}))\\ +2\rho (a_{1}^{L}+\beta_{1}(a_{1}^{U}-a_{1}^{L}))(k(\theta^{L}+\beta_{1}(\theta^{U}-\theta^{L}))-(b^{U}+\beta_{2}(b^{U}-b^{L})))\\ +2p_{1}(b^{U}+\beta_{1}(b^{U}-b^{L}))(b^{U}+\beta_{2}(b^{U}-b^{L}))-k\rho (\theta^{L}+\beta_{1}(\theta^{U}-\theta^{L}))(a_{1}^{L}+\beta_{1}(a_{1}^{U}-a_{1}^{L})) \end{array}\right) \end{array}\right)}{4{\left(k(\theta^{L}+\beta_{1}(\theta^{U}-\theta^{L}))-(b^{U}+\beta_{2}(b^{U}-b^{L}))\right)}^{2}}.$$ (26)

Let ∂*π*_*r*_/∂*p*_1_ = 0, we can obtain:

$$p_{32}^{*}=\frac{\left(\begin{array}{l} \rho (a_{1}^{L}+\beta_{1}(a_{1}^{U}-a_{1}^{L}))(k(\theta^{L}+\beta_{1}(\theta^{U}-\theta^{L}))-(b^{U}+\beta_{2}(b^{U}-b^{L})))+\\ \frac{\left(\begin{array}{l} k(\theta^{L}+\beta_{1}(\theta^{U}-\theta^{L}))(\left(\theta^{L}+\beta_{1}(\theta^{U}-\theta^{L}))+(\theta^{L}+\beta_{2}(\theta^{U}-\theta^{L})\right))\\ -(k(\theta^{L}+\beta_{1}(\theta^{U}-\theta^{L}))+(b^{U}+\beta_{1}(b^{U}-b^{L})))(b^{U}+\beta_{2}(b^{U}-b^{L})) \end{array})(\begin{array}{l} \left(1-\rho )(a_{2}^{L}+\beta_{2}(a_{2}^{U}-a_{2}^{L})\right)\\ +k\rho (a_{1}^{L}+\beta_{1}(a_{1}^{U}-a_{1}^{L})) \end{array}\right)}{k(k(b^{U}+\beta_{1}(b^{U}-b^{L}))-2(\theta^{L}+\beta_{1}(\theta^{U}-\theta^{L}))-(\theta^{L}+\beta_{2}(\theta^{U}-\theta^{L})))+2(b^{U}+\beta_{2}(b^{U}-b^{L}))} \end{array}\right)}{\left(\theta^{L}+\beta_{1}(\theta^{U}-\theta^{L}))(\left(\theta^{L}+\beta_{2}(\theta^{U}-\theta^{L}))+k(b^{U}+\beta_{1}(b^{U}-b^{L})\right))-2(b^{U}+\beta_{1}(b^{U}-b^{L}))(b^{U}+\beta_{2}(b^{U}-b^{L})\right)}.$$ (27)

By introducing Eq (27) into Eq (24), it can be solved that

$$p_{31}^{*}=\frac{\left(\begin{array}{l} \rho (a_{1}^{L}+\beta_{1}(a_{1}^{U}-a_{1}^{L}))\left(\begin{array}{l} \left(\theta^{L}+\beta_{2}(\theta^{U}-\theta^{L})\right)\\ -k(b^{U}+\beta_{1}(b^{U}-b^{L})) \end{array}\right)\left(\begin{array}{l} 2(\theta^{L}+\beta_{1}(\theta^{U}-\theta^{L}))k+k(\theta^{L}+\beta_{2}(\theta^{U}-\theta^{L}))\\ -k^{2}(b^{U}+\beta_{2}(b^{U}-b^{L}))-2(b^{U}+\beta_{2}(b^{U}-b^{L})) \end{array}\right)\\ +\left(\begin{array}{l} (b^{U}+\beta_{1}(b^{U}-b^{L}))\left(\begin{array}{l} k(\theta^{L}+\beta_{2}(\theta^{U}-\theta^{L}))-k^{2}(b^{U}+\beta_{2}(b^{U}-b^{L}))\\ -4(b^{U}+\beta_{2}(b^{U}-b^{L})) \end{array}\right)\\ +(\theta^{L}+\beta_{1}(\theta^{U}-\theta^{L}))(\left(\theta^{L}+\beta_{2}(\theta^{U}-\theta^{L}))+3k(b^{U}+\beta_{1}(b^{U}-b^{L})\right)) \end{array}\right)\left(\begin{array}{l} \left(1-\rho )(a_{2}^{L}+\beta_{2}(a_{2}^{U}-a_{2}^{L})\right)\\ +k\rho (a_{1}^{L}+\beta_{1}(a_{1}^{U}-a_{1}^{L})) \end{array}\right) \end{array}\right)}{2\left(\begin{array}{l} k\left(\begin{array}{l} -2(\theta^{L}+\beta_{1}(\theta^{U}-\theta^{L}))\\ -(\theta^{L}+\beta_{2}(\theta^{U}-\theta^{L}))\\ +k(b^{U}+\beta_{1}(b^{U}-b^{L})) \end{array}\right)\\ +2(b^{U}+\beta_{2}(b^{U}-b^{L})) \end{array}\right)(\begin{array}{l} (\theta^{L}+\beta_{1}(\theta^{U}-\theta^{L}))\left(\begin{array}{l} \left(\theta^{L}+\beta_{2}(\theta^{U}-\theta^{L})\right)\\ +k(b^{U}+\beta_{1}(b^{U}-b^{L})) \end{array}\right)\\ -2(b^{U}+\beta_{1}(b^{U}-b^{L}))(b^{U}+\beta_{2}(b^{U}-b^{L})) \end{array})}.$$ (28)

When $\frac{\partial^{2}\pi_{r}}{\partial p_{1}^{2}}=\frac{\left(\begin{array}{l} (\theta^{L}+\beta_{1}(\theta^{U}-\theta^{L}))\left(\begin{array}{l} 2(b^{U}+\beta_{2}(b^{U}-b^{L}))(\theta^{L}+\beta_{2}(\theta^{U}-\theta^{L}))-k{\left(\theta^{L}+\beta_{2}(\theta^{U}-\theta^{L})\right)}^{2}\\ +k(b^{U}+\beta_{1}(b^{U}-b^{L}))(k^{2}(b^{U}+\beta_{1}(b^{U}-b^{L}))+6(b^{U}+\beta_{2}(b^{U}-b^{L}))) \end{array}\right)\\ +2(b^{U}+\beta_{1}(b^{U}-b^{L}))(b^{U}+\beta_{2}(b^{U}-b^{L}))\left(\begin{array}{l} k(\theta^{L}+\beta_{2}(\theta^{U}-\theta^{L}))-k^{2}(b^{U}+\beta_{1}(b^{U}-b^{L}))\\ -2(b^{U}+\beta_{2}(b^{U}-b^{L})) \end{array}\right)\\ -2k{\left(\theta^{L}+\beta_{1}(\theta^{U}-\theta^{L})\right)}^{2}(\left(\theta^{L}+\beta_{2}(\theta^{U}-\theta^{L}))+k(b^{U}+\beta_{1}(b^{U}-b^{L})\right)) \end{array}\right)}{2{\left(k(\theta^{L}+\beta_{1}(\theta^{U}-\theta^{L}))+(b^{U}+\beta_{2}(b^{U}-b^{L}))\right)}^{2}}<0$ is established, $p_{31}^{*}$ and $p_{32}^{*}$ are at their maximum values, indicating that the retailer’s Stackelberg strategy is the optimal value strategy.

### 3.4 VN decision structure

The VN Decision Structure model is a representation of a non-cooperative game among participants in the market. It is based on the assumption that each competitor is fully aware of the equilibrium solution of the opponent. Under this framework, both manufacturers and retailers make independent decisions regarding wholesale prices, online channel prices, and retail prices. The Vertical Nash strategy is then employed to determine the optimal value of the decision variable.

Find the first-order conditions for the manufacturer’s profit Eq (8) regarding *p*_2_ and the retailer’s profit Eq (9) regarding *p*_1_. The following equation can be obtained:

$$\frac{\partial \pi_{m}}{\partial p_{2}}=(\begin{array}{l} \left(1-\rho )(a_{2}^{L}+\beta_{2}(a_{2}^{U}-a_{2}^{L}))+p_{1}(\theta^{L}+\beta_{2}(\theta^{U}-\theta^{L}))-kp_{1}(b^{U}+\beta_{1}(b^{U}-b^{L})\right)\\ +2p_{2}(k(\theta^{L}+\beta_{1}(\theta^{U}-\theta^{L}))-(b^{U}+\beta_{2}(b^{U}-b^{L})))+k\rho (a_{1}^{L}+\beta_{1}(a_{1}^{U}-a_{1}^{L})) \end{array}),$$ (29)

$$\frac{\partial \pi_{r}}{\partial p_{1}}=\rho (a_{1}^{L}+\beta_{1}(a_{1}^{U}-a_{1}^{L}))+p_{2}(\theta^{L}+\beta_{1}(\theta^{U}-\theta^{L}))+(b^{U}+\beta_{1}(b^{U}-b^{L}))(kp_{2}-2p_{1}).$$ (30)

Let ∂*π*_*r*_/∂*p*_1_ = 0 and ∂*π*_*m*_/∂*p*_2_ = 0, it can be solved that

$$p_{41}^{*}=\frac{\left(\begin{array}{l} \rho (a_{1}^{L}+\beta_{1}(a_{1}^{U}-a_{1}^{L}))(2(b^{U}+\beta_{2}(b^{U}-b^{L}))-k(\left(\theta^{L}+\beta_{2}(\theta^{U}-\theta^{L}))-k(b^{U}+\beta_{1}(b^{U}-b^{L})\right)))\\ +(1-\rho )(a_{2}^{L}+\beta_{2}(a_{2}^{U}-a_{2}^{L}))(\left(\theta^{L}+\beta_{2}(\theta^{U}-\theta^{L}))+k(b^{U}+\beta_{1}(b^{U}-b^{L})\right)) \end{array}\right)}{\left(\begin{array}{l} \left(k(b^{U}+\beta_{1}(b^{U}-b^{L}))+(\theta^{L}+\beta_{2}(\theta^{U}-\theta^{L})))(k(b^{U}+\beta_{1}(b^{U}-b^{L}))-(\theta^{L}+\beta_{2}(\theta^{U}-\theta^{L}))\right)\\ +4(b^{U}+\beta_{1}(b^{U}-b^{L}))(\left(b^{U}+\beta_{2}(b^{U}-b^{L}))+k(\theta^{L}+\beta_{2}(\theta^{U}-\theta^{L})\right)) \end{array}\right)},$$ (31)

$$p_{42}^{*}=\frac{\rho (a_{1}^{L}+\beta_{1}(a_{1}^{U}-a_{1}^{L}))(\left(\theta^{L}+\beta_{2}(\theta^{U}-\theta^{L}))+k(b^{U}+\beta_{1}(b^{U}-b^{L})\right))+2(1-\rho )(a_{2}^{L}+\beta_{2}(a_{2}^{U}-a_{2}^{L}))(b^{U}+\beta_{1}(b^{U}-b^{L}))}{\left(\begin{array}{l} 4(b^{U}+\beta_{1}(b^{U}-b^{L}))(\left(b^{U}+\beta_{2}(b^{U}-b^{L}))-k(\theta^{L}+\beta_{1}(\theta^{U}-\theta^{L})\right))+\\ \left(k(b^{U}+\beta_{1}(b^{U}-b^{L}))+(\theta^{L}+\beta_{1}(\theta^{U}-\theta^{L})))(k(b^{U}+\beta_{1}(b^{U}-b^{L}))-(\theta^{L}+\beta_{2}(\theta^{U}-\theta^{L}))\right) \end{array}\right)}.$$ (32)

From $p_{41}^{*}$ and $p_{42}^{*}$ being greater than 0, we know that

$$4(b^{U}+\beta_{1}(b^{U}-b^{L}))(\begin{array}{l} \left(b^{U}+\beta_{2}(b^{U}-b^{L})\right)\\ -k(\theta^{L}+\beta_{1}(\theta^{U}-\theta^{L})) \end{array})+(\begin{array}{l} k(b^{U}+\beta_{1}(b^{U}-b^{L}))+\\ \left(\theta^{L}+\beta_{1}(\theta^{U}-\theta^{L})\right) \end{array})(\begin{array}{l} k(b^{U}+\beta_{1}(b^{U}-b^{L}))-\\ \left(\theta^{L}+\beta_{2}(\theta^{U}-\theta^{L})\right) \end{array})>0\;\mathrm{and}$$

$$(\begin{array}{l} \rho (a_{1}^{L}+\beta_{1}(a_{1}^{U}-a_{1}^{L}))(2(b^{U}+\beta_{2}(b^{U}-b^{L}))-k(\left(\theta^{L}+\beta_{1}(\theta^{U}-\theta^{L}))-k(b^{U}+\beta_{1}(b^{U}-b^{L})\right)))\\ +(1-\rho )(a_{2}^{L}+\beta_{2}(a_{2}^{U}-a_{2}^{L}))(\left(\theta^{L}+\beta_{1}(\theta^{U}-\theta^{L}))+k(b^{U}+\beta_{1}(b^{U}-b^{L})\right)) \end{array})>0.$$

In addition, due to $\partial^{2}\pi_{r}/\partial p_{1}^{2}=-2(b^{U}+\beta_{1}(b^{U}-b^{L}))<0$, when the condition $\partial^{2}\pi_{m}/\partial p_{2}^{2}=$ $2(k(\theta^{L}+\beta_{1}(\theta^{U}-\theta^{L}))-(b^{U}+\beta_{2}(b^{U}-b^{L})))<0$ is satisfied, $p_{41}^{*}$ and $p_{42}^{*}$ are the equilibrium solutions of the Nash strategy.

## 4 Comparison of equilibrium solutions with different rights structures

Next, we will examine the influence of various power structures in a dual-channel supply chain on price decisions, market demand, and revenue of node enterprises in the face of uncertain demand.

When the sensitivity coefficient of channel price is given as *θ* = 0, it has an impact on the price decisions, market demand, and profits of enterprises in the dual channel supply chain nodes, based on different power structures.

**Proposition 1** In an uncertain demand environment, under the dual channel supply chain sales model, the offline channel price in the retailer-led Stackelberg game is higher than that in the Vertical Nash strategy game. The offline channel price in the retailer-led Stackelberg game is higher than that in the manufacturer-led Stackelberg game. In terms of demand, the centralized decision-making game has a higher price than the manufacturer-led Stackelberg game, and the Vertical Nash game has a higher price than the retailer-led Stackelberg game. There is a significant difference in the size relationship between obtaining equilibrium strategies for different rights structures when considering the uncertainty of market demand and not considering the impact of demand uncertainty [37–40], namely $p_{31}^{*}>p_{41}^{*}>p_{21}^{*}>p_{11}^{*}$, $d_{1}(p_{11}^{*})>d_{1}(p_{21}^{*})>$ $d_{1}(p_{41}^{*})>d_{1}(p_{31}^{*})$.

**Proof:** Firstly, we substitute *θ* = 0 into the offline retail channel price equilibrium solution Eq (13) of the centralized decision-making, the offline retail channel price equilibrium solution Eq (22) of the manufacturer Stackelberg, the offline retail channel price equilibrium solution Eq (28) of the retailer Stackelberg strategy, and the offline retail channel price equilibrium solution Eq (31) of the vertical Nash strategy, respectively. After simplification, we can obtain:

$$p_{31}^{*}-p_{41}^{*}=\frac{k^{2}(2\rho (a_{1}^{L}+\beta_{1}(a_{1}^{U}-a_{1}^{L}))(b^{U}+\beta_{2}(b^{U}-b^{L}))-k(1-\rho )(a_{2}^{L}+\beta_{2}(a_{2}^{U}-a_{2}^{L}))(b^{U}+\beta_{1}(b^{U}-b^{L})))}{2(k^{2}(b^{U}+\beta_{1}(b^{U}-b^{L}))+2(b^{U}+\beta_{2}(b^{U}-b^{L})))(k^{2}(b^{U}+\beta_{1}(b^{U}-b^{L}))+4(b^{U}+\beta_{2}(b^{U}-b^{L})))},$$ (33)

$$p_{21}^{*}-p_{41}^{*}=-\frac{k^{3}(k\rho (a_{1}^{L}+\beta_{1}(a_{1}^{U}-a_{1}^{L}))+2(1-\rho )(a_{2}^{L}+\beta_{2}(a_{2}^{U}-a_{2}^{L})))(b^{U}+\beta_{1}(b^{U}-b^{L}))}{4(k^{2}(b^{U}+\beta_{1}(b^{U}-b^{L}))+2(b^{U}+\beta_{2}(b^{U}-b^{L})))(k^{2}(b^{U}+\beta_{1}(b^{U}-b^{L}))+4(b^{U}+\beta_{2}(b^{U}-b^{L})))},$$ (34)

$$p_{11}^{*}-p_{21}^{*}=-\frac{k(k\rho (a_{1}^{L}+\beta_{1}(a_{1}^{U}-a_{1}^{L}))+2(1-\rho )(a_{2}^{L}+\beta_{2}(a_{2}^{U}-a_{2}^{L})))}{4k^{2}(b^{U}+\beta_{1}(b^{U}-b^{L}))+8(b^{U}+\beta_{2}(b^{U}-b^{L}))}.$$ (35)

Based on the previous parameter assumptions, it is established that $p_{31}^{*}-p_{41}^{*}>0$, $p_{21}^{*}-p_{41}^{*}<0$, and $p_{11}^{*}-p_{21}^{*}<0$. Consequently, $p_{31}^{*}>p_{41}^{*}>p_{21}^{*}>p_{11}^{*}$ can be derived.

The demand for offline retail, considering various rights structures, can be expressed as follows:

$$d_{1}(p_{41}^{*})=\frac{2\rho (a_{1}^{L}+\beta_{1}(a_{1}^{U}-a_{1}^{L}))(b^{U}+\beta_{2}(b^{U}-b^{L}))-k(1-\rho )(a_{2}^{L}+\beta_{2}(a_{2}^{U}-a_{2}^{L}))(b^{U}+\beta_{1}(b^{U}-b^{L}))}{k^{2}(b^{U}+\beta_{1}(b^{U}-b^{L}))+4(b^{U}+\beta_{2}(b^{U}-b^{L}))},$$ (36)

$$d_{1}(p_{11}^{*})=\frac{\rho (a_{1}^{L}+\beta_{1}(a_{1}^{U}-a_{1}^{L}))}{2},$$ (37)

$$d_{1}(p_{31}^{*})=\frac{2\rho (a_{1}^{L}+\beta_{1}(a_{1}^{U}-a_{1}^{L}))(b^{U}+\beta_{2}(b^{U}-b^{L}))-k(1-\rho )(a_{2}^{L}+\beta_{2}(a_{2}^{U}-a_{2}^{L}))(b^{U}+\beta_{1}(b^{U}-b^{L}))}{2k^{2}(b^{U}+\beta_{1}(b^{U}-b^{L}))+4(b^{U}+\beta_{2}(b^{U}-b^{L}))},$$ (38)

$$d_{1}(p_{21}^{*})=\frac{\rho (a_{1}^{L}+\beta_{1}(a_{1}^{U}-a_{1}^{L}))(k^{2}Z_{1}+4(b^{U}+\beta_{2}(b^{U}-b^{L})))-2k(1-\rho )(a_{2}^{L}+\beta_{2}(a_{2}^{U}-a_{2}^{L}))(b^{U}+\beta_{1}(b^{U}-b^{L}))}{4k^{2}(b^{U}+\beta_{1}(b^{U}-b^{L}))+8(b^{U}+\beta_{2}(b^{U}-b^{L}))}.$$ (39)

We can obtain:

$$d_{1}(p_{11}^{*})-d_{1}(p_{21}^{*})=\frac{k(k\rho (a_{1}^{L}+\beta_{1}(a_{1}^{U}-a_{1}^{L}))+2(1-\rho )(a_{2}^{L}+\beta_{2}(a_{2}^{U}-a_{2}^{L})))(b^{U}+\beta_{1}(b^{U}-b^{L}))}{4k^{2}(b^{U}+\beta_{1}(b^{U}-b^{L}))+8(b^{U}+\beta_{2}(b^{U}-b^{L}))},$$ (40)

$$d_{1}(p_{21}^{*})-d_{1}(p_{41}^{*})=\frac{k^{2}(b^{U}+\beta_{1}(b^{U}-b^{L}))(2\rho (a_{1}^{L}+\beta_{1}(a_{1}^{U}-a_{1}^{L}))(b^{U}+\beta_{2}(b^{U}-b^{L}))-k(1-\rho )(a_{2}^{L}+\beta_{2}(a_{2}^{U}-a_{2}^{L}))(b^{U}+\beta_{1}(b^{U}-b^{L})))}{2(k^{2}(b^{U}+\beta_{1}(b^{U}-b^{L}))+2(b^{U}+\beta_{2}(b^{U}-b^{L})))(k^{2}(b^{U}+\beta_{1}(b^{U}-b^{L}))+4(b^{U}+\beta_{2}(b^{U}-b^{L})))},$$ (41)

$$d_{1}(p_{41}^{*})-d_{1}(p_{31}^{*})=\frac{k^{3}(k\rho (a_{1}^{L}+\beta_{1}(a_{1}^{U}-a_{1}^{L}))+2(1-\rho )(a_{2}^{L}+\beta_{2}(a_{2}^{U}-a_{2}^{L}))){\left(b^{U}+\beta_{1}(b^{U}-b^{L})\right)}^{2}}{4(k^{2}(b^{U}+\beta_{1}(b^{U}-b^{L}))+2(b^{U}+\beta_{2}(b^{U}-b^{L})))(k^{2}(b^{U}+\beta_{1}(b^{U}-b^{L}))+4(b^{U}+\beta_{2}(b^{U}-b^{L})))}.$$ (42)

According to the previous parameter assumptions, it is known that $d_{1}(p_{11}^{*})-d_{1}(p_{21}^{*})>0$, $d_{1}(p_{21}^{*})-d_{1}(p_{41}^{*})>0$ and $d_{1}(p_{41}^{*})-d_{1}(p_{31}^{*})>0$. Therefore, $d_{1}(p_{11}^{*})>$ $d_{1}(p_{21}^{*})>d_{1}(p_{41}^{*})>d_{1}(p_{31}^{*})$ can be obtained. Similarly, Propositions 2 and 3 can be proven.

**Proposition 2** In the dual channel supply chain sales model with uncertain demand, the online channel price in the Stackelberg strategy led by retailers is higher than the online channel price in the Stackelberg strategy led by manufacturers. Additionally, the relative size of channel prices in other structures is affected by the share of demand ρ (0 < ρ < 1) that goes to traditional offline consumption channels. Similarly, the online channel demand in the manufacturer-led Stackelberg strategy is higher than that in the retailer-led Stackelberg strategy, namely $p_{31}^{*}>p_{21}^{*}$ and $d_{1}(p_{21}^{*})>d_{1}(p_{31}^{*})$.

**Proposition 3** The profitability of a dual channel supply chain operating under uncertain demand can be described by the following relationships. The profit of the centralized decision-making supply chain is greater than the profit of the vertical Nash supply chain. The profit of the centralized decision-making supply chain is also greater than the profit of the manufacturer-led Stackelberg supply chain. Furthermore, the profit of the centralized decision-making supply chain is greater than the profit of the retailer-led Stackelberg strategy. In summary, the profit hierarchy of the dual channel supply chain, from highest to lowest, is as follows: $\pi_{1c}^{*}>\pi_{4c}^{*}>\pi_{3c}^{*}>\pi_{2c}^{*}$.

When the sensitivity coefficient of channel price is given as 0<*θ*≤1, it affects the price decisions, market demand, and profits of dual channel supply chain node enterprises. The presence of numerous fuzzy variables in the obtained equilibrium solution makes comparative analysis more challenging. Hence, this paper will employ numerical simulation to investigate the subject further.

## 5 Numerical analysis and discussion

Next, a sensitivity study of the proposed model will be conducted through numerical examples to examine the psychological indicators, channel price sensitivity, market size, and other related variables of decision-makers under different power structures.

### 5.1 Numerical examples

In order to investigate the price coordination problem of dual channel supply chain products under uncertain demand, we will utilize numerical methods. Due to the challenges associated with collecting real industry data, a portion of the data is sourced from previously published literature [27], while the remaining data is hypothetical. In this study, the modified data should align as closely as possible with the dataset provided in previous studies. However, due to differing assumptions and limitations, it is not feasible to utilize the exact same dataset. Therefore, the parameter dataset considered in this study is as follows: the potential demand of the predicted market is represented by variable *a*_*i*_ = [50,60], the sensitivity coefficient of channel prices is represented by variable *θ* = [0.4,0.5], the sensitivity coefficient of self-prices is represented by variable *b* = [0.6,0.7], and the mentality indicator of each member in the dual channel supply chain is set to 0.5, denoted as variable *β*_*i*_ = 0.5. Additionally, the share of demand flowing to traditional offline consumption channels is represented by variable *ρ* = 0.55, and the coefficient between wholesale and retail prices is represented by variable *k* = 0.8.

### 5.2 The impact of variables on decision results and sensitivity analysis

**Fig 2:**
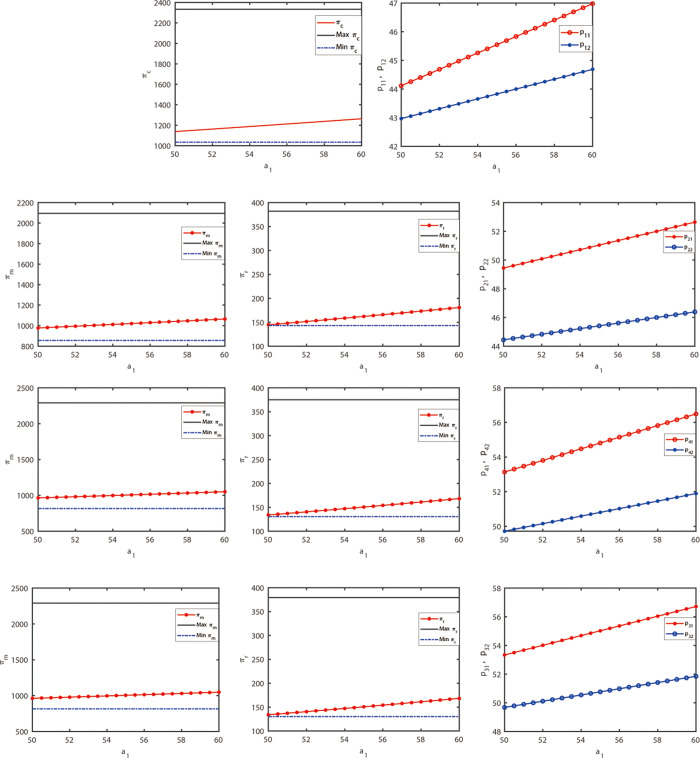
Sensitivity analysis of basic parameters of market demand.

This section aims to transform the model into a deterministic fuzzy model by specifying the constraints of fuzzy variables in the model. When the upper and lower limits of each interval are identical, the interval value model can be regarded as a brittleness model. When the lower end is equal to the upper end, the center of the interval is equal to both ends. Therefore, all demand and profit functions of interval value models can be applied to a deterministic fuzzy model. In the previous section, we obtained the optimal interval of the profit function through analytical and numerical methods. Now, if any parameter value is selected from the aforementioned range, the profit must fall within the optimal range. Due to the involvement of numerous parameters in this model, the analysis and explanation become excessively complex. However, numerical examples such as Fig 2A can be utilized to demonstrate that profits always fall within the optimal profit range. Consequently, it can be concluded that under the interval value model, the current results remain stable for a deterministic fuzzy model. For research purposes, the profit function and channel price decision variables were calculated and plotted by setting ρ = 0.55 and *k* = 0.8, while changing one parameter at a time and keeping the other parameters constant. Subsequently, discussions were also conducted on the decision results and sensitivity when the fuzzy variables *a*_1_, *b* and *θ* were altered, respectively.

First, based on the results obtained in the previous section and the given assumed parameter data, the optimal values of decision variables under different power structures in the dual-channel supply chain are shown in Table 1 when the demand is uncertain. It can be observed that the nature of the profit curve is consistent with previous literature (see Fig 2). Therefore, the assumed dataset is acceptable.

**Table 1:** The optimal value of decision variables for different rights structures in a dual channel supply chain under uncertain demand (Results preserved to 2 decimals).

	$p_{i1}^{*}$	$p_{i2}^{*}$	*d*_1_	*d*_2_	Manufacturer(*π*_*m*_)	Retailer(*π*_*r*_)	Total(*π*_*c*_)
CP	46.98	44.69	[12.49,27.16]	[10.01,23.68]	[893.84,2028.91]	[140.25,304.94]	[1034.09,2333.85]
MS	52.64	46.39	[9.21,24.61]	[11.08,25.49]	[855.88,2095.62]	[142.99,382.16]	[998.86,2477.77]
RS	56.72	51.85	[8.54,24.9]	[8.89,24.25]	[815.22,2289.91]	[130.1,379.3]	[945.32,2669.21]
VN	56.49	51.89	[8.71.,25.05]	[8.77,24.11]	[816.9,2291.13]	[130.5,375.21]	[947.4,2666.33]

The demand potential of the market is represented by *a*_*i*_, while the evolution trend of profits and aggregation prices of decision-makers under different power structures is shown in Fig 2 when *a*_*i*_ changes from 50 to 60. In Fig 2, it can be observed that the profit function increases with the increase of *a*_*i*_ in A (a), B (a) and (b), C (a) and (b), D (a) and (b). Therefore, the working principle of interval valued parameter models is also applicable to deterministic fuzzy models. If the demand potential *a*_*i*_ of the market increases, the corresponding product demand in the market will also increase. Consequently, the pricing decisions of competitors will increase with the increase of *a*_*i*_, as shown in Fig 2A(b), 2B(c), 2C(c) and 2D(c).

#### 5.2.1 The impact of decision-maker mentality indicators on competitive outcomes

**Fig 3:**
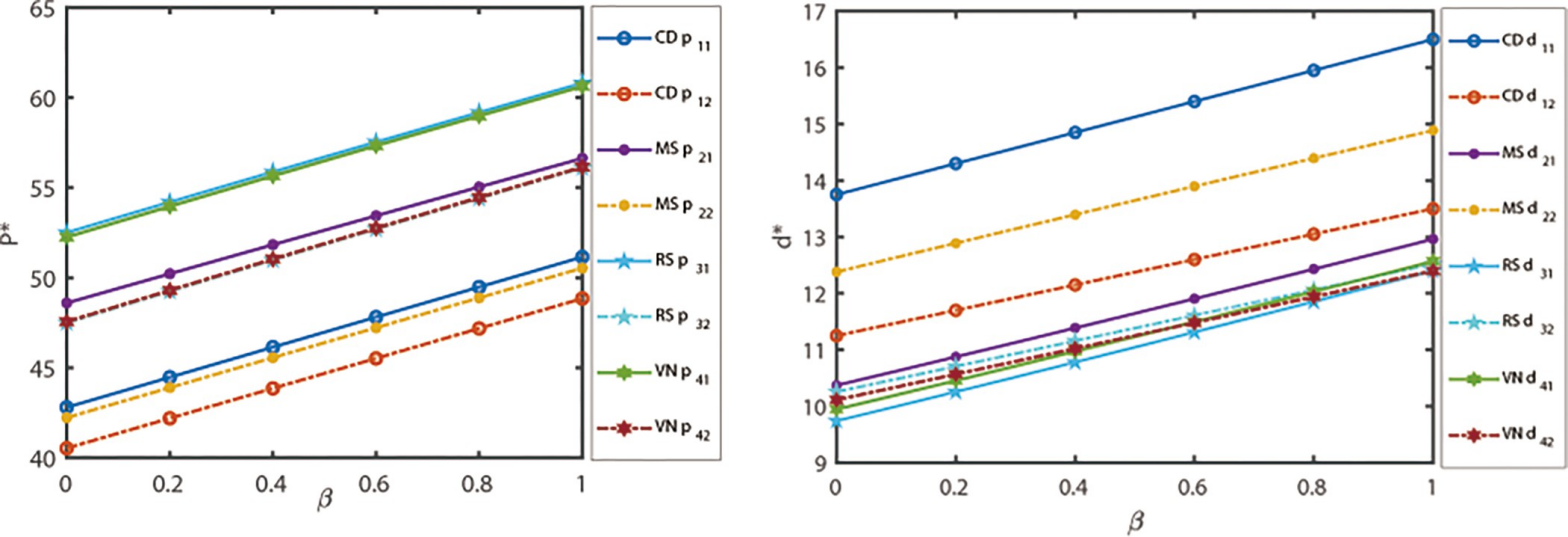
The impact of decision-maker mentality indicators on outcomes under different power structures.

The mentality indicator is a measure that reflects the risk attitude of decision-makers at different nodes within the dual channel supply chain. The risk attitude of decision-makers in node enterprises has a direct impact on the competitive outcome of dual channel supply chains. In order to analyze the impact of changes in decision-maker mentality indicators on channel prices and market demand, four different power models are considered: supply chain centralized decision-making, manufacturer Stackelberg, retailer Stackelberg strategy, and vertical Nash. Based on the parameter settings mentioned above, the following analysis will interpret the effects of these changes. Fig 3A illustrates the correlation between decision-makers’ risk attitude indicator *β* and product channel prices. It is notable that as the risk attitude indicator of decision-makers increases (indicating a more optimistic risk attitude), the prices of different product channels under the four power structure modes consistently rise. Furthermore, it is evident that online channel prices are lower compared to offline retail prices, aligning with reality. This can be attributed to the cost advantages and other factors of online channels, which indirectly demonstrates the influence of the digital economy on the real economy. Fig 3B illustrates the evolutionary correlation between risk attitude indicators of decision-makers and market demand for products. It can be observed from Fig 3B that as the risk attitude indicator of decision-makers increases (indicating a more optimistic risk attitude), the market demand for various product channels steadily rises under the four power structure models. Specifically, when centralized supply chain decision-making is employed, the demand for retail channels surpasses that of online channels. On the other hand, when either the manufacturer Stackelberg or retailer Stackelberg strategy is adopted for decision-making, the advantage of high demand in online channels becomes more prominent. In the case of vertical Nash strategy, the adoption of risk attitude indicators by decision-makers leads to a scenario where the demand for retail channels exceeds that of online channels and continues to grow. This suggests that retail channels leverage their lower retail prices to capture a larger market share, thereby increasing their own demand. Such a situation is often attributed to the promotional and other business methods employed by retail channels.

#### 5.2.2 Discussion on parameter sensitivity

In this section, we will analyze the impact of the decision-maker’s risk attitude on the evolution of decision parameters when the fuzzy variables *a*_1_, *b* and *θ* change.

**Fig 4:**
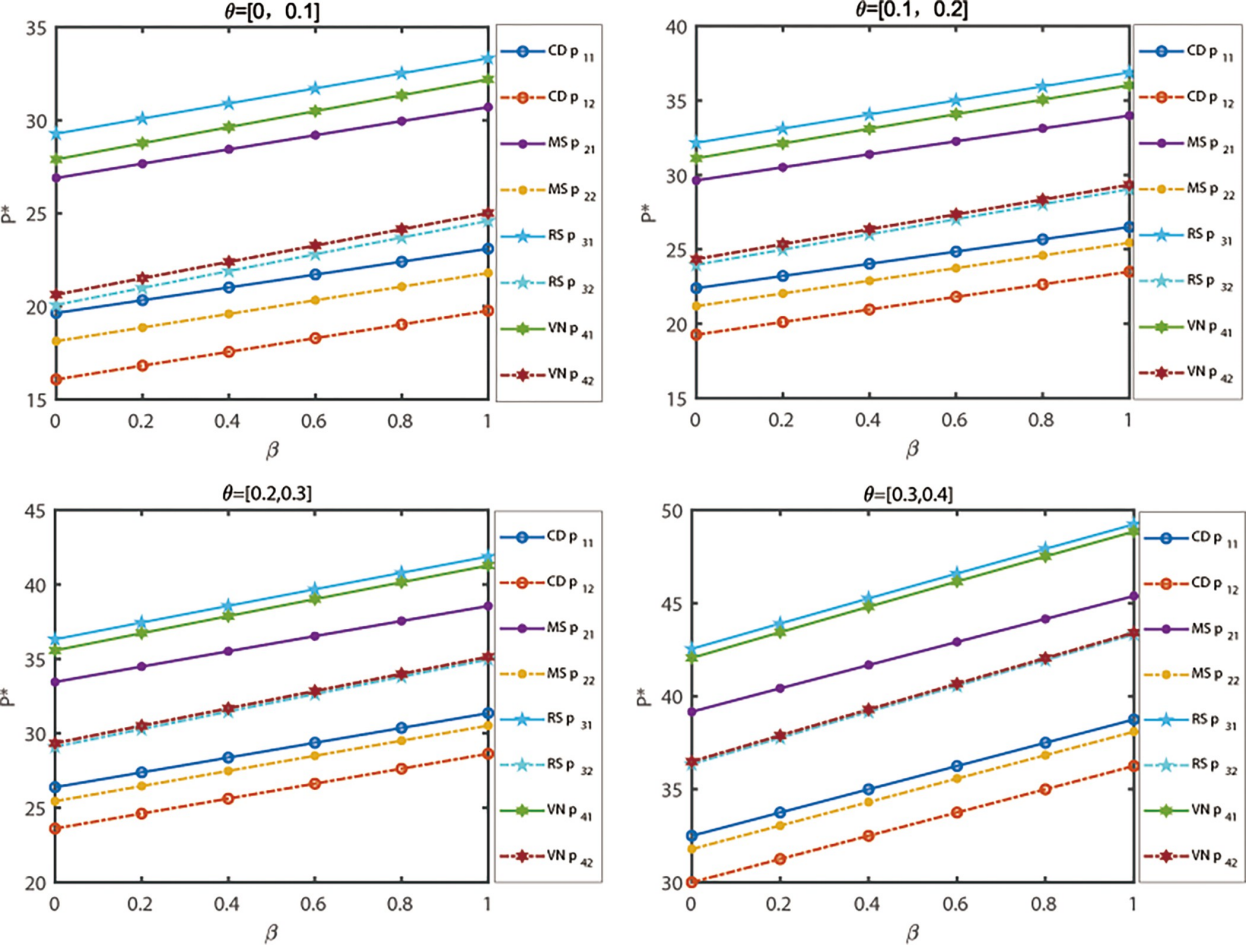
The relationship between fuzzy variables *θ* and decision-maker mentality indicators under different power structures.

To begin with, we will examine the sensitivity of different channel prices of products and decision-maker mentality indicators under different power structures, while keeping the competitor channel price sensitivity coefficients *θ* at values *θ* = [0,0.1], *θ* = [0.1,0.2], *θ* = [0.2,0.3] and *θ* = [0.3,0.4]. Fig 4 illustrates the results of this analysis. Based on Fig 3A (*θ* = [0.4,0.5]), it can be observed that when decision-makers maintain the same risk attitude, the relative price of products increases with the substitutability of products under the same mode. Furthermore, as decision-makers’ risk preferences increase, the prices of products under various power structure models show an upward trend. Additionally, the prices of retail channels are higher than those of online channels. This finding suggests that in the real economic environment, offline retail channels face a disadvantage in product price competition due to costs and other factors.

**Fig 5:**
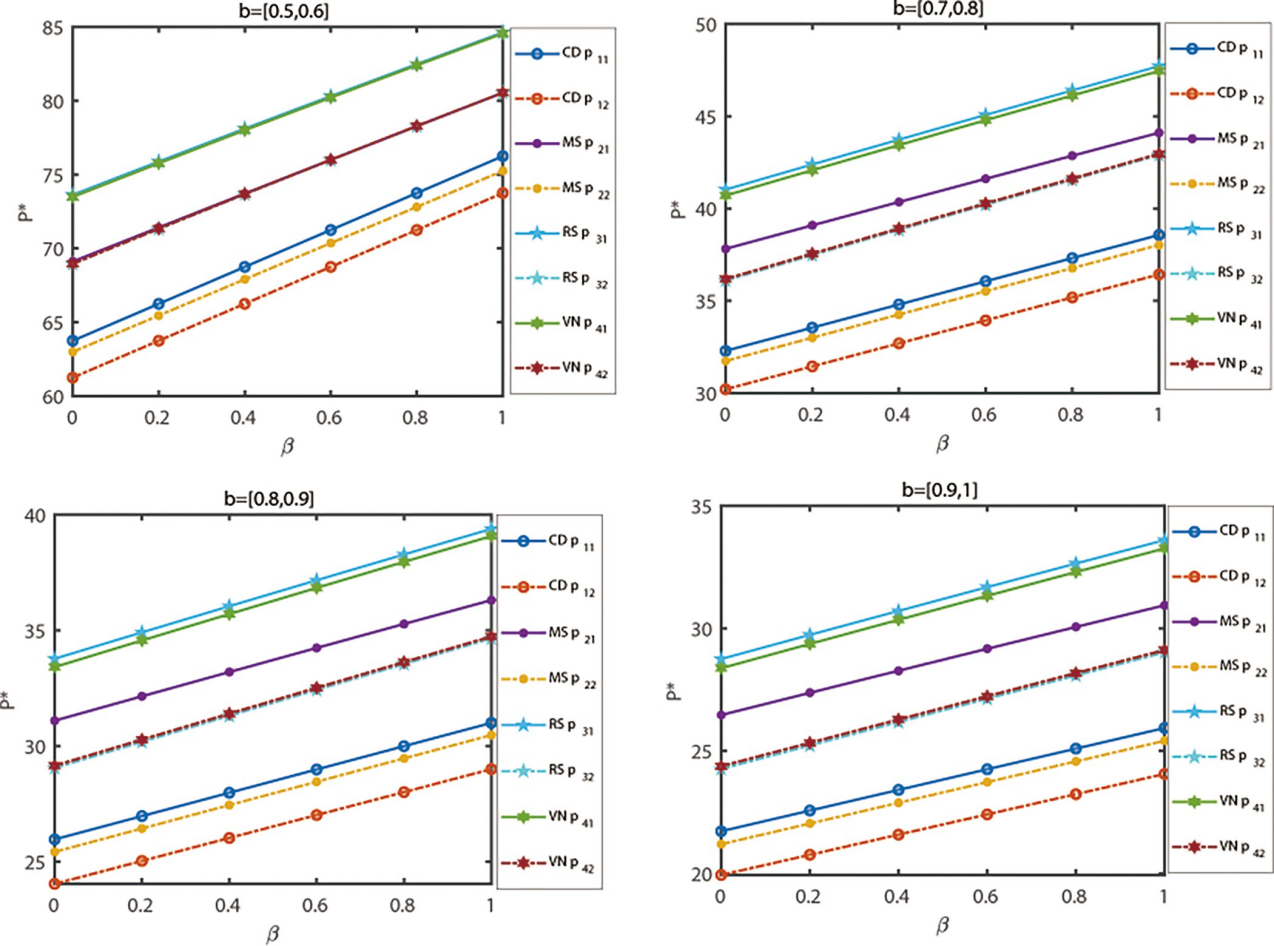
The relationship between fuzzy variables *b* and decision-maker mentality indicators under different power structures.

Fig 5 illustrates the examination of the sensitivity of product prices and decision-maker mentality indicators under different power structures, with sensitivity coefficients *b* of self-prices being *b* = [0.5,0.6], *b* = [0.7,0.8], *b* = [0.8,0.9] and *b* =[0.9,1], while keeping other parameter values constant. Referring to Fig 3A (*b* = [0.6,0.7]), the evolutionary outcomes of five decisions are presented as the sensitivity coefficient of one’s own price changes. From the evolutionary results, it can be observed that when decision-makers maintain the same risk attitude, the higher the sensitivity of market demand for price is under the same mode, the lower the price of products will be, resulting in lower profits for node enterprises. Moreover, as the risk preference of decision-makers increases, the prices of products in various power structure models exhibit a downward trend. Analysis reveals that the lower the sensitivity of market demand for price, the more likely it is for decision-makers’ risk indicators to influence decision outcomes.

**Fig 6:**
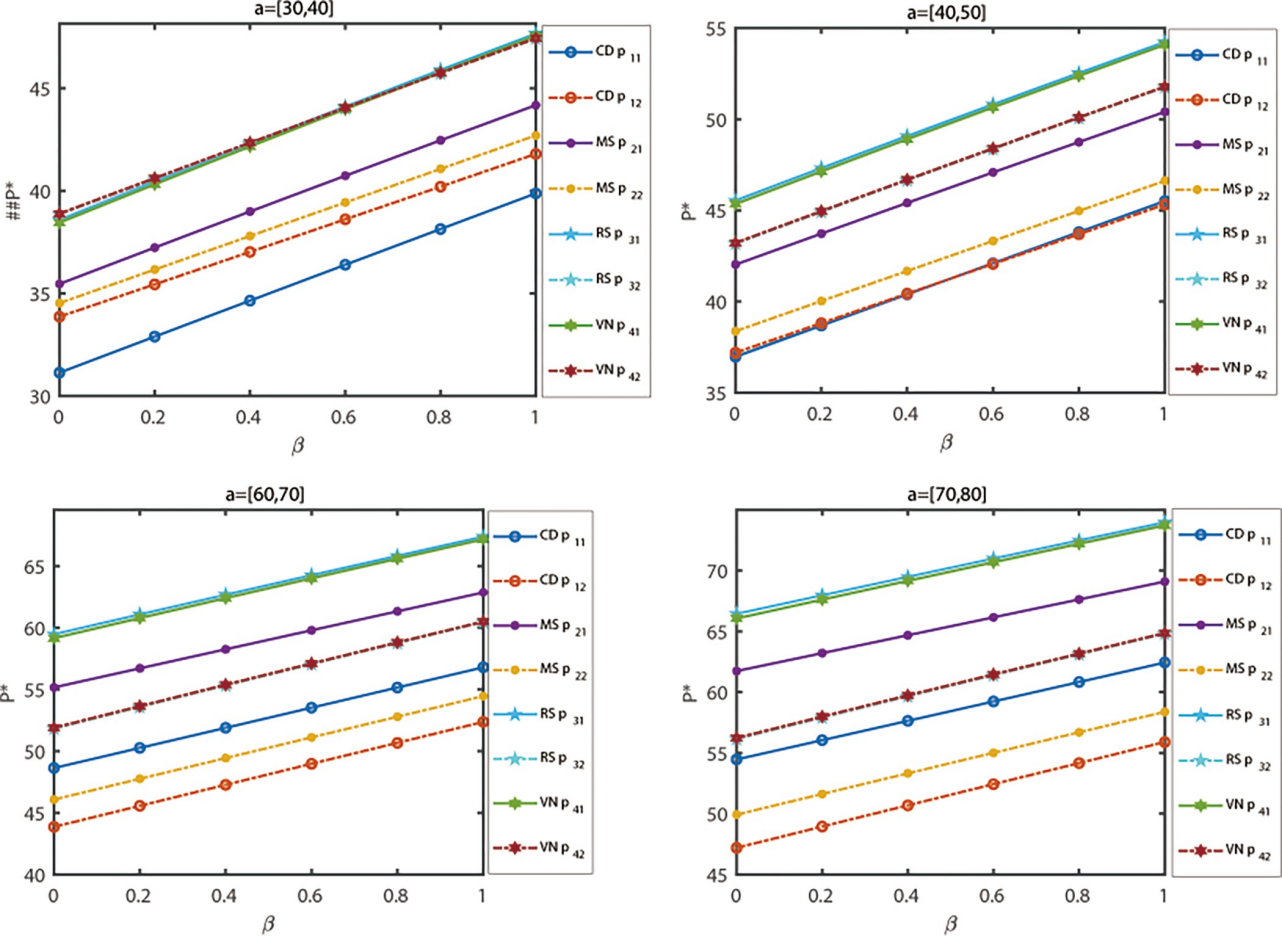
The relationship between fuzzy variables *a*_1_ and decision-maker mentality indicators under different power structures.

Fig 6 illustrates the relationship between the sensitivity of various channel prices and decision-maker mentality indicators for products under different power structures. The potential demand in the predicted market is denoted as *a*_*i*_ = [30,40], *a*_*i*_ = [40,50], *a*_*i*_ = [60,70] and *a*_*i*_ = [70,80], while other parameter values remain constant. By referring to Fig 3A (*a*_*i*_ = [50, 60]), the evolution of five decisions can be predicted when the potential demand in the market changes. The results of the evolution show that, when decision-makers maintain the same risk attitude, the price of products increases with the larger market size under the same model. Moreover, when decision-makers have a higher risk preference, the prices of products exhibit an upward trend across different power structure models. Additionally, both the market size and decision-maker risk indicators have an impact on the final decision outcome. As the market size increases, the range of obtained decision equilibrium solutions also expands. The analysis reveals that as the market size grows, the influence of decision-makers’ risk indicators on decision results diminishes.

## 6 Conclusion

This paper aims to investigate the issue of price competition in a dual supply chain, which consists of a manufacturer, a traditional offline retail channel, and an online channel, in the presence of uncertain demands. To begin with, a game model of the dual channel supply chain is constructed under various structural modes, including centralized decision-making, manufacturer Stackelberg, retailer Stackelberg strategy, and vertical Nash, taking into account the complexity characteristics of channel demand uncertainty. Furthermore, Fuzzy Theory is employed to address the uncertain demand problems in the game. By confining the demand function within an interval, an equilibrium solution for the competition in the dual channel supply chain under different power structure modes can be derived. Moreover, the impact of market size on the profits of the dual channel supply chain, customer demand, and channel prices under uncertain demand is analyzed, along with the influence of product substitutability on channel prices. Finally, the effectiveness of the proposed model is validated through numerical examples.

The research findings indicate that decision-makers’ risk attitude has a significant impact on the evolution of different channel prices in dual channel supply chains. As decision-makers become more optimistic in their risk attitude, there is a continuous increase in the trend of product prices under the four power structure models. It is observed that online channel prices are consistently lower than offline retail prices, reflecting the competitive advantage of online channels in terms of pricing. This suggests the influence of the network economy on the real economy. The study also reveals that decision-makers’ risk attitude towards dual channel supply chains can affect channel prices, demand, supply chain profits, and final decision outcomes. In smaller markets, customer demand is less sensitive to price and product substitutability is higher, indicating a greater likelihood of decision outcomes being influenced by psychological indicators. Furthermore, larger markets under the same mode result in higher product prices when decision-makers maintain the same risk attitude. Additionally, an increase in decision-makers’ risk preference leads to an upward trend in product prices under various power structure models.

While the paper offers valuable management insights, it is important to acknowledge its limitations. The paper primarily focuses on uncertainty in customer needs, with other parameters being treated as static. Future research should aim to address the competition issue in multi-channel green supply chain networks with identical decision variables. Additionally, there is potential for further exploration by incorporating multiple manufacturers producing products at different green levels and analyzing the market competition scenario. This direction presents promising opportunities for future research in the field of supply chain management.

## Supporting information

S1 Data (RAR)
